# A New Era of Data-Driven Cancer Research and Care: Opportunities and Challenges

**DOI:** 10.1158/2159-8290.CD-24-1130

**Published:** 2024-10-04

**Authors:** Felicia Gomez, Arpad M. Danos, Guilherme Del Fiol, Anant Madabhushi, Pallavi Tiwari, Joshua F. McMichael, Spyridon Bakas, Jiang Bian, Christos Davatzikos, Elana J. Fertig, Jayashree Kalpathy-Cramer, Johanna Kenney, Guergana K. Savova, Meliha Yetisgen, Eliezer M. Van Allen, Jeremy L. Warner, Fred Prior, Malachi Griffith, Obi L. Griffith

**Affiliations:** 1 Department of Medicine, Washington University School of Medicine, St Louis, Missouri.; 2 Department of Biomedical Informatics, University of Utah, Salt Lake City, Utah.; 3 Department of Biomedical Engineering, Emory University and Georgia Institute of Technology, Atlanta, Georgia.; 4 Atlanta Veterans Affairs (VA) Medical Center, Decatur, Georgia.; 5 Department of Radiology and Biomedical Engineering, University of Wisconsin, Madison, Wisconsin.; 6 William S. Middleton Memorial Veterans Affairs (VA) Healthcare, Madison, Wisconsin.; 7 Departments of Pathology and Laboratory Medicine, Indiana University School of Medicine, Indianapolis, Indiana.; 8 Departments of Radiology and Imaging Sciences, Indiana University School of Medicine, Indianapolis, Indiana.; 9 Departments of Biostatistics and Health Data Science, Indiana University School of Medicine, Indianapolis, Indiana.; 10 Departments of Neurological Surgery, Indiana University School of Medicine, Indianapolis, Indiana.; 11 Department of Computer Science, Luddy School of Informatics, Computing, and Engineering, Indiana University, Indianapolis, Indiana.; 12 Department of Health Outcomes & Biomedical Informatics, University of Florida, Gainesville, Florida.; 13 Department of Radiology, University of Pennsylvania School of Medicine, Philadelphia, Pennsylvania.; 14 Department of Oncology and Applied Mathematics & Statistics, Johns Hopkins Medicine, Baltimore, Massachusetts.; 15 Department of Ophthalmology, University of Colorado School of Medicine, Aurora, Colorado.; 16 Technology Research Advocacy Partnership, National Cancer Institute, Bethesda, Maryland.; 17 Department of Pediatrics, Harvard Medical School, Boston, Massachusetts.; 18 Boston Children’s Hospital, Boston, Massachusetts.; 19 Department of Biomedical and Health Informatics, University of Washington, Seattle, Western Australia.; 20 Department of Medicine, Dana-Farber Cancer Institute, Harvard School of Medicine, Boston, Massachusetts.; 21 Broad Institute, Cambridge, Massachusetts.; 22 Parker Institute for Cancer Immunotherapy, San Francisco, California.; 23 Departments of Medicine and Biostatistics, Brown University, Providence, Rhode Island.; 24 Lifespan Cancer Institute, Rhode Island Hospital, Providence, Rhode Island.; 25 Department of Biomedical Informatics, University of Arkansas for Medical Sciences, Little Rock, Arkansas.

## Abstract

People diagnosed with cancer and their formal and informal caregivers are increasingly faced with a deluge of complex information, thanks to rapid advancements in the type and volume of diagnostic, prognostic, and treatment data. This commentary discusses the opportunities and challenges that the society faces as we integrate large volumes of data into regular cancer care.

## Introduction

Cancer remains a major health problem. The risk for developing cancer between ages 0 and 74 years is approximately 20% (1). Almost all of us will, at some point, face a cancer diagnosis, either personally or in a close friend or family member. Cancer is a complex and heterogeneous disease. People with cancer, along with their formal and informal caregivers are increasingly faced with a deluge of sophisticated information, tests, and treatment options. Much of this complexity arises from numerous technologic advances in imaging, genomics, electronic health data mining, artificial intelligence (AI), and more. These advances have been driven by the scale and scope of the data collected and have provided tremendous support toward improving treatments, outcomes, and the quality of life of people with cancer. However, they also pose new and unique challenges, transforming the study and treatment of cancer into a case of analyzing and managing large and complex datasets.

**Figure 1.**
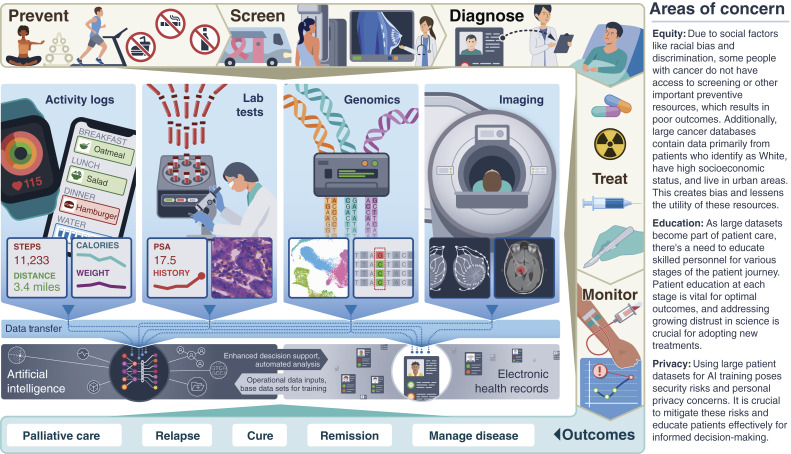
The comprehensive journey of cancer care: from prevention to personalized treatment in the era of integrated data analysis. The journey of a person with cancer begins with preventive and screening measures to avoid the development of cancer and to detect cancer at an early stage when it is more treatable. If screening detects cancer, the disease is further diagnosed and assessed *via* laboratory tests, genomic analysis, and imaging. Following diagnosis, a course of treatment and monitoring is proposed. Cancer treatment and management has a range of outcomes and often involves iterative rounds of analysis and monitoring. As the patient journey continues, outcomes that can be experienced include remission, cure, relapse, or palliative care when treatments are no longer effective. Data collected from people with cancer in each stage of the journey: prevention, screening, diagnosis, treatment, disease management, and outcome, comprise the landscape of data that are increasingly integrated into medical health records and analyzed with AI tools. The goal of the data integration is to optimize treatment to address the specific disease biology experienced by the person with cancer in a framework of personalized medicine. However, this integrated data-driven approach presents new equity, education, and privacy challenges that must be addressed in order to maximize benefits for society.

Every person with cancer faces unique challenges and opportunities related to data, and these have become much more pronounced in recent years (Fig. 1). For example, consider a patient with advanced prostate cancer. In the past decades, their care typically involved careful monitoring of a single serum biomarker (prostate-specific antigen, or PSA) for tracking treatment response. Treatment was limited to hormonal deprivation, systemic chemotherapy, and palliative corticosteroids. Today, significant advances across multiple areas have created rich datasets that have dramatically changed clinical management. Prostate cancer management still requires longitudinal serum PSA monitoring but may now also include: (i) laboratory tests for general assessments of kidney and liver function; (ii) expanded germline and somatic molecular data obtained from tumor and blood sources to guide use of targeted therapies in clinical trials and molecular disease monitoring; (iii) advanced use of histopathologic images (images of cancer tissue) to improve traditional diagnostic and prognostic methods (e.g., Gleason grading; iv) integration of different imaging techniques (e.g., CT, multiparametric MRI, nuclear imaging, etc.) across multiple time points to help with disease diagnosis, monitoring, and risk assessment for surgery or radiation; and (v) patient reported data on interventions occurring outside of clinical environments, such as dietary or exercise interventions, sometimes measured using personal health devices. All of these data modalities must be combined and considered as part of the electronic health record (EHR) and shared between patients and providers. Increasingly, each new data modality and its integration may benefit from advanced AI approaches, which can identify valuable but unrecognized signals in data and potentially automate laborious and error-prone tasks with improved accuracy. Importantly, as it gains widespread adoption and regulatory approval, this integration of data streams requires regular cross-evaluations from multidisciplinary specialists (e.g., surgeons, medical oncologists, nuclear medicine physicians, nurse practitioners, pathologists, laboratory geneticists, imaging technologists, informatics specialists, and scientists). The integration of data streams enables new levels of personalized medicine, as opposed to the “one size fits all” approach of the past.

Given this complex and interconnected landscape, effective cancer management now depends on building and supporting informatics technology including new algorithms, software, databases, and, critically, experts trained in their application. In this commentary, we discuss the opportunities and challenges for the training of experts, policies, investment, and open science approaches for cancer informatics to maximize the benefits of data-driven cancer care.

## Opportunities and Early Successes Arising from Data-Driven Cancer Research and Care

After sequencing of the first human genome in 2001, attention was focused on sequencing of cancer genomes. This work resulted in large datasets like The Cancer Genome Atlas (TCGA), the study of which has identified many new genetic variants associated with specific cancers.

Tumors often have large number of variants (alterations in DNA, often referred to as mutations), which number from tens to many thousands. Some of these variants have been shown to affect crucial genes and drive cancer development, whereas the majority are uncharacterized and/or do not have a known impact on tumor function. A major challenge is to identify and define which variants are the key drivers for a given tumor, because these variants can often be targeted with specialized therapies that can be more effective and have fewer side effects than chemotherapy. For example, lung cancer patients with variants in a gene called *EGFR* or certain gene fusions involving the *ALK* gene benefit from targeted treatment with tyrosine kinase inhibitors. In breast cancer, the adoption of targeted treatments like ERBB2 (commonly called HER2) inhibitors such as trastuzumab, PI3K inhibitors, and CDK4/6 inhibitors have demonstrated strong benefits. One report found that trastuzumab increased the 5-year survival in HER2 positive breast cancer patients from only 2% of patients to 31% (2). This is a rare win in a field that regularly struggles to balance efficacy and toxicity.

Medical imaging is an essential component of the cancer journey (Fig. 1). Imaging techniques like annual mammograms and CT screening for lung cancer allow physicians to visualize and characterize many types of cancer. Imaging has long been a major tool to both diagnose and confirm a diagnosis, as well as track how a patient is responding to therapy. In a recent clinical trial, PET imaging was used to monitor patient response to therapy, enabling researchers to show that chemotherapy could be replaced with safer targeted options without loss of treatment efficacy (3). Imaging is also used to plan and monitor both surgical and radiotherapy. Increasingly, the medical image quality is enhanced by AI that is built into the imaging equipment, often reducing the amount of time the patient must spend in the scanner. Preliminary work indicates that in some cancers, AI-assisted image analysis can help predict which tumors will respond to certain drugs (4). AI tools also help radiologists and pathologists to reach more accurate diagnoses by providing suggestions and recommendations based on extensive automated analyses of the large amount of imaging data produced by modern medical imaging devices.

With the implementation of relevant US Federal regulations in the mid-2010s, the adoption of EHR technology increased drastically creating a situation in which nearly all US healthcare settings use EHR systems. EHR systems serve as a record of all interactions between patients and healthcare systems, including a wide variety of data that can provide documentation of treatments, and also be used for both cancer prevention and care when cancer occurs. This data includes demographics, social history (e.g., tobacco/alcohol use, social determinants of health), family health history, preexisting or cooccurring conditions, laboratory test results, imaging, medications, referrals, and clinical notes. EHR systems also include clinical decision support tools that can be used to help improve cancer care such as healthcare provider reminders for preventive care (e.g., breast and colorectal cancer screening, HPV vaccination), patient engagement portals and communication tools such as electronic decision aids (e.g., shared decision-making for lung cancer screening), and telehealth visits. EHRs have also been able to drive population-based cancer prevention through algorithms that identify eligible patients, coupled with digital- and human-based patient outreach interventions connecting patients with services such as tobacco cessation treatment, lifestyle programs, cancer screening, and genetic testing for hereditary cancer syndromes (5).

AI is defined as the ability of a machine to perform complex tasks that typically require human intelligence. The machine does this by learning patterns from representative examples and employs this information for decision-making on new unseen data. In the last decade, the unprecedented growth in the field of AI has presented an extremely impactful opportunity to improve cancer outcomes. These opportunities are due to the confluence of several important developments. First and foremost, the explosion in patient data on a macro- (e.g., MRI or CT images), micro- (e.g., digitized histopathologic slides), and nano-scale (e.g., DNA changes in a tumor) is an important paradigm shift. This explosion of data has led to the collection of population health-level data on a routine basis during cancer treatments. The second important paradigm shift is the increased availability of low-cost, high-quality computational resources across the world; however, the extent to which these resources are available varies. The increased accessibility of computational resources coupled with advances in AI is expected to dramatically reduce the workload for human experts (e.g., radiologists, pathologists, surgeons, oncologists), and improve clinical decision-making while also providing tumor-specific personalized information (6). For example, traditional radiographic evaluation of tumors relies upon largely *qualitative* features from clinical images. However, computational imaging and computational/digital pathology have enabled obtaining *quantitative*, objective tumor measurements capturing tumor heterogeneity (different lineages of cancer cells in the same tumor). These AI-driven biomarkers have been evaluated for improved prognosis, prediction of response to a specific therapy, as well as assessment of tumor response. Further promise comes from the adoption of large-scale, pretrained deep-learning models, often referred to as foundation models.

## Emerging Social Challenges of Large Datasets in Cancer Research and Treatment

Translation of basic science advances into clinical applications remains a major challenge. The landscape of data created by new technologies is noisy and complex. This adds to the difficulty of predicting which efforts will translate into meaningful changes in cancer screening and treatment. Additionally, efforts to translate data to clinical practice are labor intensive and the outcome is uncertain, making the decision to embark on translation efforts challenging. Although sequencing human and tumor genomes led to early optimism, the progress establishing a clear translational effect has been slow. The discovery of variants in the *BRCA1*/*BRCA2* genes are a seminal example of how genomic studies were translated into successful cancer screening and treatment protocols. *BRCA1*/*BRCA2* genes encode proteins that play a central role in DNA repair, cell-cycle control, and chromosomal stability. Since the discovery of these genes and their relationship to breast cancer risk in the early 1990s, genetic screening for variants in these genes is now recommended for those meeting the criteria including family and personal history of cancer, and certain ethnic groups. Additionally, tumors with *BRCA1*/*BRCA2* variants receive specific therapies including alkylating agents (e.g., cisplatin) and PARP inhibitors.

Despite the successful translation of genomic discoveries in some cancers, basic discoveries in many other cancers have not had the same translational success. For example, admixture mapping analyses have identified chromosomal locus *8q24* as a region of the genome that confers increased risk to prostate cancer in men of West African ancestry. A recent review (7) suggests that as the amount of African ancestry increases in this region of the genome, so does the risk for prostate cancer. This admixture mapping result has been replicated in a large genome wide association study focused on men of African descent. Indeed, this region has been comprehensively shown to confer risk for glioma, and breast, colorectal, bladder, and stomach cancers (8), as well as familial prostate cancer (9). However, because the underlying biological mechanisms by which germline variation in this genomic region modulates risk for cancer remain unknown (10), there are still no clear risk assessment tools or clearly defined recommendations based on variation in this genomic region. This is just one of many examples in which the research community struggles to find translational effect for compelling computational results.

In addition, the sheer abundance of data presents a new challenge. For example, tools for analysis and interpretation of cancer genome data have lagged well behind the generation of large cancer genomic datasets like TCGA. The deluge of cancer variants identified creates a significant interpretation bottleneck for our cancer healthcare system (11). Similar interpretation bottlenecks arise from increasingly high throughput imaging technologies, large EHR datasets, and other data streams. This abundance of data remains a challenge without appropriate tools and trained professionals for its cataloging, harmonization, and interpretation. Potential solutions to these problems include curated databases that house clinical information on cancer variants and AI classification systems, all of which rely on some degree of human oversight and expertise. Cancer research and care have been transformed and at times drastically improved by the abundance of new and large data modalities. However, it is equally important to understand that without deliberate interventions large cancer datasets exacerbate social challenges associated with the privacy, health disparities, and the unequal access to data-informed care.

## Challenges Related to Health Equity

In a perfect world, new tools and technologic resources would have an equalizing effect creating a rising tide that floats all boats. However, as in many other fields, healthcare data are plagued with several types of biases that result from and exacerbate health disparities. Data-driven approaches have historically relied on data from high-resource academic medical centers, which disproportionately provide care to patients who are White, have high socioeconomic status, and live in urban areas. As a result, medical knowledge derived from these data will disproportionately benefit those patients. Additionally, even when diverse groups of patients are represented in healthcare datasets, disparities in healthcare utilization due to social determinants of health, such as lack of health insurance and low health literacy, lead to information presence bias (i.e., certain groups having disproportionately more comprehensive and more accurate healthcare data than others). As a result, benefits that depend on those data such as participation in clinical trials and personalized care will be disproportionately offered to patients who have better data.

Recent studies have raised concerns about the use of data variables such as race in prediction models that aim to assess patient risk and identify patients who may benefit from certain types of care. For example, a recent article published in the *New England Journal of Medicine* listed 13 widely used clinical prediction models, including three in cancer that use race as a variable to adjust the prediction score without a plausible biological reason (12). Although well-intentioned, race-adjustment in these models function as a proxy for social determinants of health, changing the prediction output and directing care away from those patients.

The developers of clinical prediction models should be encouraged to further employ methods to identify prediction performance disparities and adopt deliberate approaches to mitigate those disparities. The expensive genetic testing, AI, and EHRs needed for modern care are disproportionately available in high-resource academic medical centers and tertiary healthcare delivery networks. Although modern data-driven approaches have an enormous potential to transform cancer care, they can also cause profound exacerbation of health disparities. Therefore, deliberate approaches are crucial to ensure equity and fairness in data-driven cancer care, as well as accounting for potential morphologic and molecular differences in the cancer phenotype across different populations. For example, it has been shown that computational pathologic tools can identify subtle morphologic differences in the appearance of prostate and endometrial cancer between African American and European American cancer patients. When population-specific models are used for predicting cancer outcomes, they perform significantly better than population agnostic models (13, 14). These findings suggest that when AI models are aware of population-level differences, through the assessment of large-scale diverse multi-institutional data, more accurate and generalizable predictions across patient populations can be achieved. To this end, international healthcare consortia based on either data sharing or federated learning are essential, especially for rare cancers, and can address the concern that AI models do not effectively interpret variation across human populations (15).

## Conclusion

Realizing the potential of large data to maximize benefits while minimizing harms and challenges is not a small endeavor. The application of large data to cancer diagnosis, care, and treatment has been facilitated by the development of robust computational capabilities and infrastructure, and biotechnology that has advanced our abilities to observe phenotypes and describe underlying mechanisms that drive cancer. We now have the ability to collect precise measurements of disease and we are working to create tools that will harness these data in ways that were not imaginable 50 years ago. Additionally, new treatments for cancer target specific disease processes and minimize systemic harm. These advances in treatment are exemplified by targeted therapies like imatinib and trastuzumab, which act against BCR–ABL and ERBB2 cancer variants. The presence of these variants in tumors was assessed in widely used genomic tests that are used to make treatment decisions. Because of these technologic successes, imatinib and trastuzumab are now considered essential medicines by the WHO and are produced as generic or biosimilar compounds. These biosimilar compounds not only benefit industrialized nations but also provide a global reach and can begin to solve the overwhelming problem of cancer beyond industrialized nations, in which more than half of the cancer cases occur. Additionally, deliberate resource allocation can address disparities head-on. Grants for genetic research focused on underserved populations, and curation projects to build out publicly available datasets reflecting diverse populations will facilitate progress toward equitable science and patient care. In parallel, free and open knowledgebases and educational resources can further spread the benefits of large data utilization in cancer and provide opportunities for greater participation and innovation.

## References

[1] Mattiuzzi C , Lippi G. Current cancer epidemiology. J Epidemiol Glob Health 2019;9:217–22. 31854162 10.2991/jegh.k.191008.001 PMC7310786

[2] Sundquist M , Brudin L, Tejler G. Improved survival in metastatic breast cancer 1985–2016. Breast 2017;31:46–50. 27810699 10.1016/j.breast.2016.10.005

[3] Pérez-García JM , Cortés J, Ruiz-Borrego M, Colleoni M, Stradella A, Bermejo B, . 3-year invasive disease-free survival with chemotherapy de-escalation using an ^18^F-FDG-PET-based, pathological complete response-adapted strategy in HER2-positive early breast cancer (PHERGain): a randomised, open-label, phase 2 trial. Lancet 2024;403:1649–59. 38582092 10.1016/S0140-6736(24)00054-0

[4] Koh D-M , Papanikolaou N, Bick U, Illing R, Kahn CE Jr, Kalpathi-Cramer J, . Artificial intelligence and machine learning in cancer imaging. Commun Med 2022;2:133. 36310650 10.1038/s43856-022-00199-0 PMC9613681

[5] Del Fiol G , Kohlmann W, Bradshaw RL, Weir CR, Flynn M, Hess R, . Standards-based clinical decision support platform to manage patients who meet guideline-based criteria for genetic evaluation of familial cancer. JCO Clin Cancer Inform 2020;4:1–9. 31951474 10.1200/CCI.19.00120 PMC7000231

[6] Pinto-Coelho L . How artificial intelligence is shaping medical imaging technology: a survey of innovations and applications. Bioengineering (Basel) 2023;10:1435. 38136026 10.3390/bioengineering10121435 PMC10740686

[7] Johnson JR , Woods-Burnham L, Hooker SE Jr, Batai K, Kittles RA. Genetic contributions to prostate cancer disparities in men of West African descent. Front Oncol 2021;11:770500. 34820334 10.3389/fonc.2021.770500 PMC8606679

[8] Tong Y , Tang Y, Li S, Zhao F, Ying J, Qu Y, . Cumulative evidence of relationships between multiple variants in 8q24 region and cancer incidence. Medicine (Baltimore) 2020;99:e20716. 32590746 10.1097/MD.0000000000020716 PMC7328976

[9] Dupont WD , Breyer JP, Plummer WD, Chang SS, Cookson MS, Smith JA, . 8q24 genetic variation and comprehensive haplotypes altering familial risk of prostate cancer. Nat Commun 2020;11:1523. 32251286 10.1038/s41467-020-15122-1 PMC7089954

[10] Matejcic M , Saunders EJ, Dadaev T, Brook MN, Wang K, Sheng X, . Germline variation at 8q24 and prostate cancer risk in men of European ancestry. Nat Commun 2018;9:4616. 30397198 10.1038/s41467-018-06863-1 PMC6218483

[11] Krysiak K , Danos AM, Kiwala S, McMichael JF, Coffman AC, Barnell EK, . A community approach to the cancer-variant-interpretation bottleneck. Nat Cancer 2022;3:522–5. 35624339 10.1038/s43018-022-00379-w PMC9872366

[12] Vyas DA , Eisenstein LG, Jones DS. Hidden in plain sight - reconsidering the use of race correction in clinical algorithms. N Engl J Med 2020;383:874–82. 32853499 10.1056/NEJMms2004740

[13] Bhargava HK , Leo P, Elliott R, Janowczyk A, Whitney J, Gupta S, . Computationally derived image signature of stromal morphology is prognostic of prostate cancer recurrence following prostatectomy in African American patients. Clin Cancer Res 2020;26:1915–23. 32139401 10.1158/1078-0432.CCR-19-2659 PMC7165025

[14] Azarianpour S , Corredor G, Bera K, Leo P, Fu P, Toro P, . Computational image features of immune architecture is associated with clinical benefit and survival in gynecological cancers across treatment modalities. J Immunother Cancer 2022;10:e003833. 35115363 10.1136/jitc-2021-003833 PMC8814810

[15] Pati S , Baid U, Edwards B, Sheller M, Wang S-H, Reina GA, . Federated learning enables big data for rare cancer boundary detection. Nat Commun 2022;13:7346. 36470898 10.1038/s41467-022-33407-5 PMC9722782

